# 885. Integrated Blood Culture and Molecular Diagnostic Device Reduces Time-to-Result to a Quarter of Traditional Methods

**DOI:** 10.1093/ofid/ofac492.077

**Published:** 2022-12-15

**Authors:** Laura Huning, Devika Varma, Abbey Jackson, Peter Bohlen, Rainer Ng, Vamsee Pamula

**Affiliations:** Baebies, Inc., Durham, North Carolina; Baebies, Inc., Durham, North Carolina; Baebies, Inc., Durham, North Carolina; Baebies, Inc., Durham, North Carolina; Baebies, Inc., Durham, North Carolina; Baebies, Inc., Durham, North Carolina

## Abstract

**Background:**

Prolonged antibiotic exposure in infants with negative blood cultures is associated with adverse outcomes, disruption of the developing microbiome, and increased carriage of antibiotic resistance genes. However, early administration of antibiotics significantly reduces risk of infant morbidity and mortality caused by infections, and therefore faster culture-to-identification is highly desirable. We combined blood culture (BC) and PCR-based pathogen identification into a single digital microfluidic (DMF) device to speed up BC results.

**Methods:**

Our DMF BC+PCR device has a custom-built BC bottle incubator, which is connected to a disposable cartridge that performs sample preparation and PCR. Standard BC bottles were inoculated with *K. pneumoniae*, then incubated in either the DMF BC device or a traditional BC device (BD BACTEC 9050). Samples were drawn from the DMF BC bottle at 3, 4 and 5 hours and loaded in the cartridge for automated sample processing and PCR. Time-to-positivity for the DMF BC+PCR device was determined using cycle threshold and compared with the traditional method, which uses fluorescence from BC bottle followed by PCR on a separate device.

**Results:**

Bacterial presence could be detected as quickly as 3 hours post inoculation (C_t_ values of 34, 31 and 28 at 3, 4, and 5 hrs) with five replicates of *K. pneumoniae* samples on the DMF BC+PCR device (growing 10x every hr), compared to > 12 hrs for the traditional method (10.5 hr culture and 2 hr PCR identification). Additionally, for 5 common pathogens that cause neonatal sepsis (*K. pneumoniae, E. coli, S. aureus, S. agalactiae,* or *S. epidermidis*), we also demonstrated that the DMF BC bottle incubator and the traditional BC device show similar growth curves using optical methods to determine growth.

**Conclusion:**

The DMF BC system yields bacterial growth rates equivalent to traditional methods, but can drastically reduce time to pathogen identification, from 12 hrs to 3 hrs by leveraging PCR sensitivity to determine growth rather than fluorescence in the BC bottles. To our knowledge, this is the first demonstration of using an integrated microfluidic PCR platform to speed up culture and identification. Further work is ongoing to include antibiotic susceptibility testing and expand the panel of pathogens detected on the same cartridge.

**Disclosures:**

**Laura Huning, PhD**, Baebies, Inc.: Stocks/Bonds **Devika Varma, PhD**, Baebies, Inc.: Stocks/Bonds **Abbey Jackson, PhD**, Baebies, Inc.: Stocks/Bonds **Peter Bohlen, n/a**, Baebies, Inc.: Stocks/Bonds **Rainer Ng, PhD**, Baebies, Inc.: Stocks/Bonds **Vamsee Pamula, PhD**, Baebies, Inc.: Stocks/Bonds.

**Figure 1.**
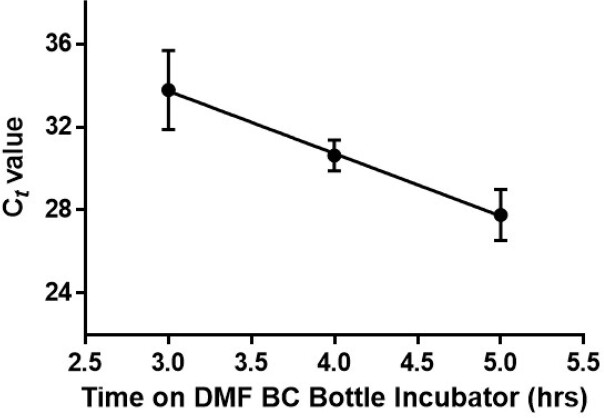
Five replicates of K. pneumoniae were incubated on the DMF BC+PCR device. We observed Ct values of 34, 31, and 28 at 3, 4, and 5 hours post inoculation. Error bars denote reproducibility over all 5 replicates at each time point.

**Figure 2.**
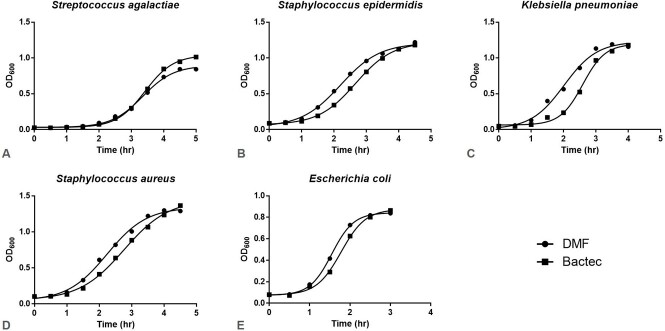
Duplicates of S. agalactiae, S. epidermidis, K. pneumoniae, S. aureus and E. coli were incubated on the DMF BC+PCR device and compared to duplicates of the same sample incubated on a comparator incubator. Growth curves on DMF BC+PCR incubator are similar to those from the comparator.

